# Systematic review of hematopoietic stem cell transplantation in patients with ataxia telangiectasia: a case report and an individual patient-level analysis

**DOI:** 10.3389/fimmu.2026.1754093

**Published:** 2026-04-13

**Authors:** Hayrunnisa Bekis Bozkurt, Virginia Meda Spaccamela, Moustapha Hassan, Matthias Felber, Ulrike Zeilhofer, Aida Zeckanovic, Jean-Pierre Bourquin, David Jacquier, Francesco Ceppi, Fabio Candotti, Tayfun Güngör

**Affiliations:** 1 Department of Pediatrics, Division of Pediatric Allergy and Immunology, Umraniye Research and Training Hospital, İstanbul, Türkiye; 2 Department of Stem Cell Transplantation, Zürich University, Children’s Hospital, Zürich, Switzerland; 3 Department of Laboratory Medicine, Division of Biomolecular and Cellular Medicine, Karolinska Institutet, Stockholm, Sweden; 4 Department of Oncology, Zürich University, Children’s Hospital, Zürich, Switzerland; 5 Department of Pediatrics, Division of Hematology and Oncology, Vaud University Hospital Center, Lausanne, Switzerland; 6 Department of Internal Medicine, Division of Immunology, Vaud University Hospital Center, Lausanne, Switzerland

**Keywords:** ataxia telangiectasia, drug monitoring, immunodeficiency, malignancy, stem cell transplantation

## Abstract

**Background:**

There is a lack of clinical data on hematopoietic stem cell transplantation (HSCT) in Ataxia-Telangiectasia (A-T) patients due to the underlying chromosomal instability that leads to low tolerance to chemotherapy. To effectively manage cancer and immune risks, there is a need for improved HSCT protocols, novel therapies, and long-term monitoring. This report describes a 16-year-old boy with A-T and T-ALL who achieved long-term leukemia-free survival after HSCT using a tailored, drug-monitored conditioning regimen. His results were analyzed in the context of a systematic review of the literature on HSCT outcomes in A-T patients.

**Methods:**

A thorough literature review was conducted using a comprehensive search of the PubMed, Scopus, and Google Scholar databases. The search was limited to studies published between September 1, 2000, and September 1, 2025. Eligible studies were required to involve human participants and to include at least one patient with a confirmed diagnosis of A-T, with transplantation interventions.

**Results:**

The analysis included 16 A-T patients, including our patient, who underwent HSCT. The median age at transplantation was 48 months (interquartile range [IQR]: 22–142 months). Myeloablative conditioning (MAC) was administered to two patients, both of whom died. Reduced-intensity conditioning (RIC) was utilized for nine patients, with three deaths (33.3%). Reduced-toxicity conditioning (RTC) was administered in two patients, with one patient experiencing a fatal outcome. In total, eight patients (50%) experienced significant drug-related toxicities, eight (50%) had GvHD and only eight patients (50%) survived. Our patient underwent HSCT of a matched sibling donor after administration of adjusted treosulfan doses (cumulative AUC of 4671 mg/Lxh), and achieved leukemia-free survival with complete hematological and normalized thymic function without graft-versus-host disease (GvHD).

**Conclusion:**

Despite the historically poor survival outcomes observed in transplanted A-T patients, new HSCT strategies, such as treosulfan therapeutic drug monitoring and personalized drug profiles to select potent but less toxic agents, warrant reevaluation to achieve durable remission in leukemia and lymphoma. These findings underscore the necessity to persist in the development of innovative HSCT approaches with the objective of expanding therapeutic options for both malignancies and combined immunodeficiency.

## 1 Introduction

Ataxia-telangiectasia (A-T) is a rare, autosomal recessively inherited primary immunodeficiency disorder. The condition is caused by mutations in the ATM (Ataxia-Telangiectasia Mutated) gene which is located on chromosome 11q23 (1). This gene produces a serine/threonine protein kinase, which plays a central role in regulating the cellular DNA damage response, particularly in the recognition and repair of double-strand DNA lesions induced by ionizing radiation. ATM phosphorylates a network of downstream substrates (p53, BRCA1, and CHK2, etc.) to regulate cell-cycle checkpoint control, genomic integrity maintenance, and programmed cell death through activation (2, 3). Biallelic loss-of-function mutations in this gene have been shown to result in clinical symptoms, including multisystem neurodegenerative disorder. This disorder is characterized by progressive cerebellar ataxia, oculocutaneous telangiectasias, immunodeficiency, marked radiosensitivity, and an increased predisposition to malignancy (3, 4). The neurological deterioration associated with A-T is primarily due to the selective apoptosis of Purkinje and granule cells in the cerebellum, as well as the degeneration of spinal and other neuronal pathways. These pathological changes are attributed to the cumulative effects of unrepaired DNA damage, oxidative stress, and defective neuronal homeostasis resulting from ATM deficiency in A-T patients (1).

The estimated incidence of this condition is approximately 1 in 100,000 live births. In Europe, however, the rate is closer to 1 in 150,000 (5). Children with A-T have an elevated risk of developing cancers, particularly non-Hodgkin’s lymphoma and T-cell acute lymphoblastic leukemia (T-ALL). In classical A-T cases, life expectancy in classical A-T cases is typically confined to a range of 15 and 25 years, with the most prevalent causes of mortality being cancer and progressive lung disease (5, 6). Overall life expectancy has improved due the increased use of antibiotics and immunoglobulin replacement therapy (IgRT). However, early malignancy remains a therapeutic dilemma. Additionally, although dexamethasone-loaded erythrocyte infusion offers promising outlook, neurological deterioration persists over time, albeit at a decelerated rate (7). Currently, there is no known cure for A-T, and treatment remains predominantly supportive (1).

Although hematopoietic stem cell transplantation (HSCT) has emerged as a potential therapeutic approach, its utilization remains controversial due to significant risks associated with the procedure (8). The sensitivity of tissues to chemotherapeutic agents, including alkylating drugs such as busulfan and treosulfan, as well as to radiation, largely depends on cell proliferation rate and DNA repair capacity. Tissues, such as bone marrow and gastrointestinal mucosa, which rapidly divide, are particularly vulnerable to cytotoxic damage induced by these treatments. Dose-limiting toxicities are readily apparent in HSCT conditioning regimens.

There is limited clinical knowledge regarding this treatment, and only a few cases of A-T that have undergone HSCT have been documented in the literature (8, 9). Due to chromosomal instability, patients with A-T exhibit a reduced tolerance to chemotherapy, and clinical data on treatment outcomes in surviving patients are limited as well (9). A-T continues to pose significant cancer and immune risks in children, underlining the need for better HSCT protocols, new drug strategies, enhanced cancer monitoring, and long-term follow-up for growth and nutrition. This report presents a case of long-term survival in a 16-year-old boy with both A-T and mixed phenotype-ALL. The patient underwent a matched sibling donor HSCT after a tailored conditioning regimen using therapeutic drug monitoring (TDM) of treosulfan, fludarabine and rabbit Anti-T-lymphocyte globulin. It also provides a comprehensive systematic overview and analysis of the transplant outcomes of A-T patients in the literature.

## 2 Patient characteristics and follow-up period

A 16-year-old A-T patient from a consanguineous family with a history of recurrent upper respiratory tract infection and leukemia is described. At the time of admission to our clinic, the patient was 12 years old and exhibited developmental delays, characterized by dystonic choreiform movements, behavioral disorders, and stunted growth. These symptoms were accompanied by radiological findings that revealed signs of delayed bone age. He also had conjunctival telangiectasia and increased serum AFP levels. Molecular analysis revealed the presence of a homozygous mutation in the ATM gene (*607585), c. 6188G>A; p.Gly2063Glu. A family genetic consultation was conducted, revealing that both parents were heterozygous carriers of the variant in the ATM gene. At the age of 12, he was admitted to the hospital with dyspnea and mediastinal mass. The patient has been diagnosed with mixed phenotype ALL with a NUP214-ABL1 translocation. He received corticosteroid therapy and chemotherapy in accordance with established induction and consolidation according to AIEOP-BFM 2017 protocol. On the 16th day of treatment, there was evidence of more than 50% infiltration of the blasts into the bone marrow, and the mediastinal mass had decreased by only 50%. Given that the *in vitro* drug profile analysis of the leukemic blasts demonstrated resistance against all standard chemotherapies, but good sensitivity to tyrosine-kinase inhibitors (10), Ponatinib 15 mg was added to the treatment plan: Phase M (Vincrystine) + Ponatinib 30 mg (MTX HD 1: 2 gr/m2, HD2: 5 gr/m2, HD3: 5 gr/m2). The patient was recommended for HSCT for induction-refractory mixed phenotype leukemia and the remaining high risk of malignancies due to his immunodeficiency. He became MRD negative under Ponatinib and Phase M treatment on day 78.

He underwent allogeneic HSCT using his 6/6 HLA-matched sister as the donor. The conditioning regimen was scheduled for 3 x 12 g/m^2^ Treosulfan under therapeutic drug monitoring (TDM) with a target range of 4800–6000 mg/L x h (11). After a single-dose administration of treosulfan at 12 gr/m^2^ (infused over a period of one hour) on day -7 the calculated cumulative area of under the curve (AUC) was determined to be 4671 mg/Lxh. Consequently, the remaining two treosulfan doses were deemed unnecessary and were not administered. Fludarabin was administered at a cumulative dose of 5 x 30 mg/m^2^ on days -7 to -3 without TDM, and anti-thymocyte globulin (ATG) at a cumulative dose of 10 mg/kg on days -3 to -1 was added to prevent graft-versus-host disease (GvHD). The following drugs were used to prevent GvHD: cylosporine A, administered continuously starting on days -1 and methotrexate (MTX) on days +1, +3 and +6. Hematological engraftment of donor cells was achieved on days +27, +23, and +17 for absolute neutrophil counts (ANC >0.5 G/L), absolute lymphocyte counts (ALC >0.2 G/L), and platelets (PLT; >50 G/L), respectively (12).

**Figure 1:**
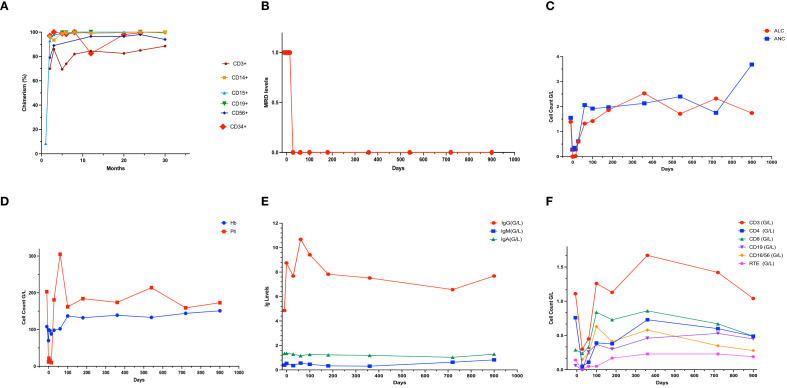
Patients’ chimerism **(A)**, MRD levels **(B)**, hematologic reconstitution **(C)** and **(D)**, immunologic reconstitution **(E)** and **(F)**. ALC, Absolute lymphocyte count; ANC, Absolute neutrophil count; Hb, Hemoglobin; Plt, Platelet; Ig, Immunoglobulin; RTE, Recent thymic emigrant cells.

After undergoing HSCT, the patient initially exhibited mixed donor chimerism which evolved into a complete donor chimerism by the 30th month after HSCT. In addition, the results demonstrated complete hematological and immune reconstitution including a normal thymic output (see **Table 1**). There were no indications of GvHD or infectious complications. His neurological development remained stable during the conditioning and engraftment processes. However, he did demonstrate a gradual decline during the post-HSCT period, necessitating assistance with ambulation. His neurologic score was 11 points before HSCT and 22 points after HSCT according to the SARA scale, with a patient-reported score of 5 points. His choreiform movements persisted, but there was no exacerbation. He has more difficulty holding a spoon. As illustrated in **Figures 1A–F**, the patient`s donor chimerism, MRD levels, and hematological and immunological reconstitution are documented.

**Table 1:** Hematological and immunologic characteristics of patient at the last visit.

Parameters	Patients value	Reference value
WBC	5.95	(4.5-12.0) (G/L)
Hb	151	(130–170) (G/L)
ALC	1.74	(1.0-5.0) (G/L)
ANC	3.68	(1.5-8.5) (G/L)
Plt	173	(150-400) (G/L)
CD3	1.04	(0.70-2.10) (G/L)
CD4	0.49	(0.30-1.30) (G/L)
CD8	0.49	(0.20-1.20) (G/L)
Naive CD4	20,21	(%) (=.23-0.77) (G/L)
γδT cells	6	(%)
CD19	0.45	(0.11-0.57) (G/L)
NK cells	0.19	(0.09-0.60) (G/L)
RTE (CD31/45RA/CD4)	0.19	(0.23-0.77) (G/L)
IgG	7.68	(7.30-12.30) (G/L)
IgM	0.82	(0.58-1.82) (G/L)
IgA	1.29	(1.09-2.74) (G/L)
IgG1	4.34	(3.70-12.80) (G/L)
IgG2	2.24	(1.06-6.10) (G/L)
IgG3	0.61	(0.18-1.63) (G/L)
IgG4	0.54	(0.035-2.30) (G/L)
Anti-Tetanus-IG	0.52	(>0.1) (IU/mL)
Anti-Pneumococcal IgG	214.33	(>28.2) (mg/L)

WBC, white blood count; Hb, Haemoglonine; ALC, Absolute lymphocte count; ANC, Absolute neutophyle count; Plt, Plateletes; RTE, recent thymic emmigrant cells; Ig, Immunoglobuline.

## 3 Materials and methods

This systematic review and individual patient data (IPD) analysis was conducted in accordance with the PRISMA 2020 statement and the PRISMA-IPD guidelines (13).

### 3.1 Eligibility criteria

For the study, only reports published in English and appearing in peer-reviewed journals were considered. The study types that met the inclusion criteria included case reports, cohort studies, and letters to the editor.

The population is as follows: The population under study had to include human subjects, including at least one patient diagnosed with A-T based on clinical and/or genetic confirmation.

Intervention: A-T patients who are scheduled to undergo or have undergone hematopoietic stem cell transplantation treatment.

Study type: Published case reports or case series.

Outcomes: The following criteria were used to determine the inclusion of studies in the review: mortality rate, GvHD rate, transplant-related complications, and neurological outcome (if possible).

The following studies were excluded from analysis: systematic and narrative reviews, editorials, commentaries, unpublished works, dissertations, government documents, books and book chapters, and conference abstracts. Studies involving animals or solely *in vitro* laboratory experiments were excluded from the analysis.

### 3.2 Literature search

For the systematic review, a comprehensive search was conducted across PubMed (https://pubmed.ncbi.nlm.nih.gov/), Scopus (www.scopus.com), and Google Scholar (https://scholar.google.com) databases. The search was conducted between September 1st, 2000, and September 1st, 2025. The search keywords included “Ataxia Telangiectasia” and “hematopoietic stem cell transplantation,” and/or “stem cell transplantation” and/or “bone marrow transplantation” as well as “ATM mutations” and “hematopoietic stem cell transplantation” and/or “stem cell transplantation” and/or “bone marrow transplantation.”.

### 3.3 Study selection

**Figure 2:**
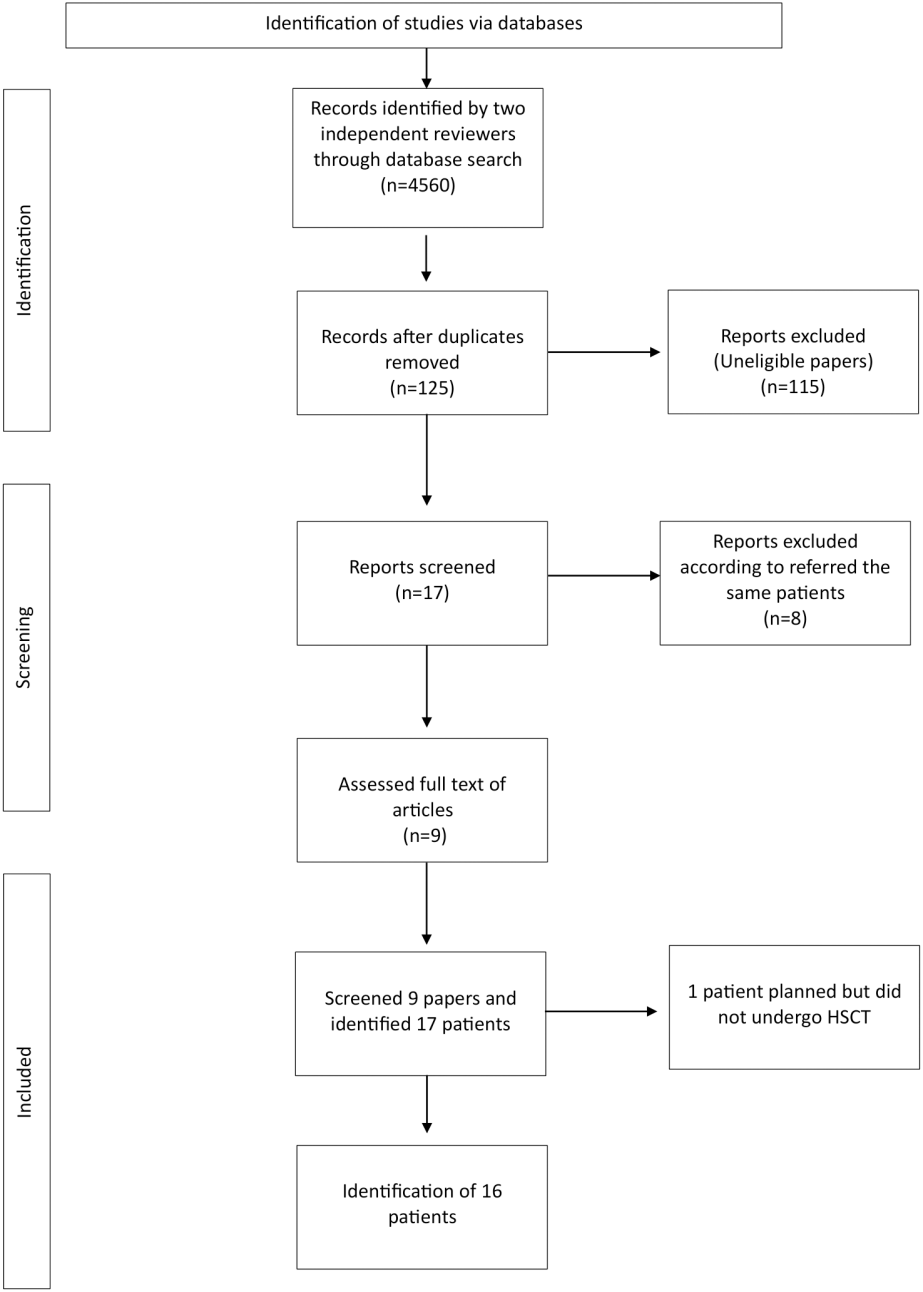
The flowchart of the study.

The selection of studies was conducted in two stages. Initially, two independent reviewers were tasked with the responsibility of screening titles and abstracts for eligibility. The full texts of potentially relevant articles were reviewed independently. Disagreements were resolved through a collaborative process involving discussion and consensus building. A comprehensive review of the relevant literature and patient records was conducted, leading to the identification of nine papers and 17 patients. One patient had been scheduled to undergo HSCT but was deferred, resulting in the analysis being performed on 16 patients. The flowchart of the study is shown in **Figure 2**.

### 3.4 The collection of individual patient

For each patient, a patient data form has been meticulously designed. The data collection form encompassed a comprehensive array of metrics, including the patient's age at the most recent visit, the duration of the follow-up period, the age at the transplantation, the patient's sex, the genetic investigations, the indication for HSCT, the donor type, the conditioning regimen, the stem cell source, the presence of acute and chronic GvHD, transplant-related complications, and the patient's status as alive or deceased. For each patient, all reports are meticulously reviewed, employing in detail using primary sources (authors, Supplementary Materials, etc.) to prevent double-counting.

### 3.5 Risk of bias

The Joanna Briggs Institute (JBI) Critical Appraisal Tools were used to assess the methodological quality of the nine included studies (14). These studies described 16 patients from nine studies, and the results showed an overall mean score of 77.3% (intermediate-high quality). However, the study`s limited sample size, the absence of a control group, clinical heterogeneity, and its retrospective and descriptive nature indicate a very low level of certainty. Despite these limitations, this study offers a comprehensive review and re-analysis of all currently published stem cell transplantation cases in Ataxia Telangiectasia, providing a structured synthesis of a rare and clinically significant therapeutic approach.

### 3.6 Statistical analysis

Statistical analysis was conducted using the SPSS software package version 25.0 for Windows (SPSS Inc., Chicago, IL, USA). The figures were created using GraphPad Prism version 9. The Kolmogorov-Smirnov test was applied to determine whether continuous variables followed a normal distribution. Data with a normal distribution were presented as the mean ± standard deviation (SD), while non-normally distributed data were shown as the median with the interquartile range (25th–75th percentile). Descriptive statistics were also carried out. Due to the absence of comparator groups and the limited number of cases, formal effect-size meta-analyses were not conducted. Instead, pooled descriptive analyses were conducted.

## 4 Results

In the literature, 17 patients were scheduled to undergo HSCT, and 16 patients actually underwent the procedure. The patient characteristics are outlined in **Table 2**. Of the patients, 11 (73.3%) were male and 4 (26.7%) were female. The median age at the time of HSCT was 48 months (interquartile range [IQR]: 22–142 months).

**Table 2:** Characteristics of patients with Ataxia Telangiectasia who underwent HSCT.

	Age/gender/clinic	Genetic analysis	Age at HSCT	Donor	Conditioning regimen	Follow-up time after HSCT	Follow-up process	Neurologic development	Outcome	Ref.
P1	32 mo Male **HyperIgM phenotype (PID)**	Homozygous mut. (exon 11) 1316 T >C/439 L >P	22 mo	BMT (MSD)	Treo 3x12 g/m2 FLU5x30 mg/m2 RATG-Fresenius 3x20 mg/kg MAC	10 mo	+d 25: GvHD skin II, liver II (controlled) + 8th mo: B cells, IgG and liver enzymes high, neutrophils low + 10th mo:fulminant hepatic failure	NA genetic studies concluded in the postmortem process	Dead	(22)
**P2**	11 yrs (2018) Male ***T-ALL***	NA	36 mo	BMT (MSD)	FLU 5x30 mg/m2) RATG-Fresenius 3x20 mg/kg) BU (oral) 0.5 mg/kg, 2x/d, total 2 mg/kg) RIC	96 mo	Mucositis (grade II) ADV, BKV and EBV-LPD (treated with cidofovir and RTX) 8 yrs: leukemia in remission Wheelchair bound No infection	Did not worsen Slowly deterioration	Alive	(25)
P3	7 yrs (2018) Female **SCID (PID)** Diagnosed at the 2 yrs after HSCT	NA	12 mo	BMT (MUD)	BU (ıv) 2x0.5 mg/kg/d) FLU 6x30 mg/m2 CY 2x20 mg/kg/d Alemtuz. 1x 0.25 and 3x 0.5 mg/kg/d RIC	60 mo	GvHD grade 1 skin (controlled with steroids) +6 mo: AIHA (steroid responsive) +8 mo: Completed T cell reconstitution 6 mo required for IgRT	Symptoms were observed in the second year of life. After 2 yrs slowly progressed	Alive	(25)
P4	4 yrs (2018) Male **Pre-emptive HSCT** Sib died of A-T and Hodgkin/HLH	NA	23 mo	BMT (MUD)	BU (ıv) 2x0.5 mg/kg/d FLU 6x30 mg/m2 CY 2x20 mg/kg/d RIC	24 mo	Uneventful 6 mo after transplantation, cyclosporine was stopped Decreasing donor chimerism to 40%	No exacerbations slowly progression	Alive	(25)
**P5**	16 yrs (2016) Male ***CD20+/CD30+ EBV pos. NHL*** (skin, ear, cervical, intrapulmonary)	Compound heterozyg. For 2 splice site mut. (IVS38-2A4G in exon 39 and c.6095G4A (R2032K) (exon 43)	13 yrs	BMT (MSD) (AT heterozyg.)	(First NHL treatment protocol applied) FLU 180 mg/m2 BU 1.6 mg/kg CY 40 mg/k) RTX 2x375 mg/m2 RIC	30 mo	VOD, BKV hemorrhagic systitis, Gİ hemorrhage). recurrent sepsis/ventilation support No acute/chronic GvHD +10 mo: CD19+/NK cells nl. +18 mo: lymphocyte/CD8+ T-cells nl. +30 mo: CD3+ and CD4+ cells nl.	Able to sit and stand without support	Alive	(21)
P6	10 yrs (2018) Male **Preemptive HSCT** only A-T phenotype and skin and joint granulomas	Two frameshift mut. (c.478_482delTCTCA, p.Ser160Alafs, and c.320delC, p.Pro1069Leufs)	4 yrs	BMT (MSD)	FLU 5x30 mg/m2/d CY 4x 20 mg/kg/d RATG-Fresenius 20 mg/kg RIC	72 mo	No acute toxicity No affect growth negatively Complete remission of skin and joint granulomas Slow increase of AFP	Milder progression of ataxic symptoms	Alive	(20)
P7	4.5 yrs Female **Rubella vaccine related granulomas on skin (PID)** A-T phenotype -diagnosed at 3 yrs	c.2098C>T, p.Gln700*; second mut. probably intronic)	4 yrs	TCRab/CD19-depleted PBSC (MUD)	FLU 5x30 mg/m2/d CY 4x 20 mg/kg/d RATG-Fresenius 20 mg/kg RIC	6 mo	Skin lesions regressed, granuloma resolved with scaring at the 10th week +d21: Skin GHVD III +5 mo: Donor chimerism100% +6 mo: Hepatic VOD and liver failure	NA	Dead	(28)
P8	6 mo (2022) Male **Pos. TREC-based NBS for SCID (no classical SCID, T-/B-cell lymphopenia) HyperIgM CID (PID) PJP at the age of 4 mo**	c.5979_5983delTAAG, p.(Ser1993Argfs*23); c.7875_7876delinsGC, p.(Asp2625_ Ala2626delinsGluPro	put on hold	-	-	-	Vitamine B3 started and HSCT planned T cell and B cell counts spontaneously increased and were in the normal ranges at the 6th months of age	NA	Alive	(31)
P9	14 mo Male CID (PID) Severe infections	NA	8 mo	BMT (MUD)	Treo 36 mg/m2 FLU 150 mg/m2 Alemtuz 1 mg/kg MAC	6 mo	Grade 1–2 skin GvHD EBV-PTLD	NA	Dead	(23)
P10	42 mo Female **Bone marrow failure**	NA	22 mo	BMT (MFD)	Treo 46g/m2 FLU 150 mg/m2 RIC	20 mo	Grade 3 liver and skin GvHD PTLD Hepatic failure	NA	Dead	(23)
P11	105 mo Female NA	NA	101 mo	BM or PBSC (MSD)	BU (dose NA) CY(dose NA) NA	4 mo	Grade 2 skin and gut Multiorgan failure	NA	Dead	(23)
P12	12.5 yrs Male **Malignancy (Type is NA)**	NA	11.5 yrs	PBSC (MFD)	FLU 150 mg/m2 CY 0.3 mg/kg RIC	12 mo	Skin GvHD (Grade NA)	NA	Dead	(23)
P13	12.3 yrs Male **Malignancy (Type is NA)**	NA	12 yrs	BMT (MSD)	BU (Dose NA) CY (Dose NA) NA	3 mo	Grade 2 skin GvHD Pericardial effusion hemorrhagic cystitis +3 mo: died	NA	Dead	(23)
P14	15.3 yrs (2018) Male **Malignancy (Type is NA)**	NA	13 yrs	HLA-identical sibling (MSD) BMT	BU 1.6 mg/kg FLU 180 mg/m2 CY 40 mg/kg RTC	27 mo	No GvHD Hemorrhagic cystitis VOD, GI bleeding	NA	Dead	(23)
P15	7 yrs (2019) Male **Preemptive** HSCT Mild recurrent infections (PID)	NA	5 yrs	BMT (MSD)	FLU (Dose NA) CY (Dose NA) RIC	24 mo	No GvHD Adequate immune reconstitution	NA	Alive	(27)
P16	18 mo (2023) NA **Newborn screning identified (SCID) (PID)**	NA	8 mo	NA	NA NA	10 mo	Clinically well (after 10 mo of HSCT)	NA	Alive	(26)
**P17 (our patient)**	16 yrs Male ***Malignancy*** T cell leukemia One year leukemıa therapy and Ponatinib	c. 6188G>A; p.Gly2063Glu	13.5 yrs	BMT (MSD)	Treo 12 g/m^2^ one dose (after TDM with an AUC: 4671 mg/Lxh) FLU 30 mg/m^2^ RATG-Fresenius 10 mg/kg RTC	30 mo	Immune and hematological reconstitution at 2 mo of age. No GvHD No infection	Very slowly progression, he can walk needing help similarly before HSCT, but has a more difficulty holding a spoon	Alive	

BMT, Bone marrow transplant; CID, Combined immunodeficiency; EBV-LD, EBV lymphoproliferative disorder; GvHD, Graft versus Host Disease; HSCT, Hematopoietic stem cell transplantation; Mo, months; MFD, Matched family donor; MSD, Matched sibling donor; MUD, Matched unrelated donor; NA, Not available; NHL, Non-hodgkin lymphoma; PBSC, Peripheric blood stem cell; PJP, Pneumocystis jirovecii Pneumonia; PTLD, Post-Transplant Lymphoproliferative Disorder; VOD, Venooclusive disease; yrs, years; BU, Alemtuz, Alemtuzumab; Busulfan; CY, Cyclophosphamide; FU, Fludarabin; Treo, Treosulfan; RATG, rabbit Anti T-lymphocyte globulin; RTX, Rituximab; TDM, Therapeutic Drug Monitoring, NBS, Newborn screening; TREC, T-cell Receptor Excision Circles, MAC, Myeloablative regimen; RIC, Reduced ıntensıty conditioning regimen; RTC, Reduced toxicity regimen.

HSCT was performed in 6 patients (37.5%) before the age of 24 months, which is the estimated onset of neurological decline, and in 10 patients (62.5%) before 60 months of age. The post-transplantation follow-up period reported in the literature was a median of 22 months (IQR: 7–30 months). The indications for transplantation included malignancies in six patients (37.5%), Rubella-induced skin granulomas in two (12.5%), severe combined immunodeficiency (SCID) phenotype in three (18.8%), bone marrow failure in one (6.3%), and pre-emptive transplantation in three (18.8%). GvHD was observed in eight patients, representing an incidence of 50%. Acute GVHD grades 1–2 occurred in five patients, representing 31.3% of the sample, while GVHD grades 3–4 occurred in two patients, representing 12.5% of the sample. In one patient, the GVHD grade is not reported. There was no reported chronic GVHD. Hepatic veno-occlusive disease and/or hepatic failure were reported in 5 patients (31.3%), 4 of whom (80%) died. It is important to note that only one patient (P5) was reported as being alive. Eight patients (50%) exhibited relevant drug-related toxicities, including one case of grade 2 mucositis, one instance of multiorgan failure, one patient requiring ventilation support, three cases of hemorrhagic cystitis, and one patient with pericardial effusion. There were no reports of kidney failure and nervous system deterioration.

At the conclusion of the study period, only eight patients (50%) survived the procedure. This process includes the mean time of approximately two years after HSCT.

Myeloablative conditioning (MAC) is a rigorous pre-transplant regimen that aims to completely eliminate the recipient’s bone marrow and immune system. This regimen includes the administration of full-dose and targeted busulfan (BU) (target cumulative AUC: 85–95 mg·h/L), fludarabine (FLU) 150–160 mg/m², or treosulfan (Treo) 30–42 g/m² combined with thiotepa 8–10 mg/kg. Conversely, a reduced-intensity conditioning (RIC) regimen constitutes a less intensive pre-transplant approach that partially suppresses the recipient`s hematopoietic system, typically employing BU (target cumulative AUC 60–70 mg·h/L), FLU150–180 mg/m², or Treo 30–42 g/m², FLU150–160 mg/m². RTC regimens were subsequently characterized as new versions of MAC with reduced toxicity. This evolution can be exemplified by the substitution of cyclophosphamide (CY) with FLU (15). MAC regimens employing the aforementioned definitions were utilized in two patients, both of whom passed away. Nine patients were administered RIC regimens, and three of them (33.3%) died. RTC regimens were administered to two patients, one of whom died.

## 5 Discussion

A-T is a multisystem disorder stemming from ATM gene mutations, affecting immune regulation, hematopoiesis, and endocrine function, and resulting in diverse clinical symptoms including progressive neurodegeneration and ataxia, for which there is currently no curative treatment (16). Given the absence of a curative treatment for A-T and the high mortality from malignancies and pulmonary complications, the question has arisen as to whether HSCT could play a role in the management of these patients suffering from intractable hematological and immunological disease (17). In 2004, HSCT was shown to enable effective engraftment, restore immune function, and prevent the onset of lymphoma in Atm-deficient mouse models using reduced intensity conditioning (RIC) (18). Furthermore, syngeneic hematopoietic stem cell transplantation (HSCT) has been associated with prolonged survival and partial neurological recovery in mice, attributed to the migration and integration of ATM-competent cells (19). In patients with A-T, only a limited number of case reports have documented successful allogeneic HSCT as part of oncological treatment protocols (20, 21). If A-T remains undiagnosed, the use of conventional conditioning regimens may lead to severe complications due to impaired DNA repair and the underlying chromosomal instability (22, 23). A recent registry-based analysis by the European Society of Bone Marrow Transplantation on chromosomal instability syndromes included eight individuals with A-T, revealing a poor overall survival rate of 25% after a median follow-up duration of 35 months (23).

Another key consideration is that A-T is an immunodeficiency disease with variable presentations. Patients with A-T often present with combined immunodeficiency, including low immunoglobulins, lymphopenia, and impaired B- and T-cell function due to defective DNA repair mechanisms (16, 24). The study revealed that one patient had a Pneumocystis jirovecii pneumonia and two had rubella-induced granulomas. These findings suggest significant T-cell dysfunction. HSCT has the potential to restore immune balance by replenishing naive T-cell populations from the thymus and thereby addressing the underlying immune defect (16). However, HSCT has not been shown to improve neurological symptoms or other systemic features of A-T. There are also risks of drug toxicity which could potentially accelerate the progression of neurological decline by causing excessive apoptosis of neurons. Therefore, a careful risk-benefit evaluation is essential. In this meta-analysis, three patients presented with severe clinical signs of immunodeficiency (ID) (P1,P3, P9), and ten patients showed immunodeficiency based on laboratory findings (22, 25, 26). Two of the three ID patients underwent myeloablative regimens and did not survive. Conversely, Duecker et al. achieved a successful transplant outcome in a 5-year-old patient with only mild recurrent infection and mild immunodeficiency symptoms (P15) using a RIC regimen (27). Similarly, successful treatment with a RIC regimen has been reported in two patients presenting with the A-T phenotype and cutaneous granulomas (P6, P7) (20, 28). Due to the encouraging outcomes, a preemptive HSCT using a RIC regimen was successfully performed in three patients (P4, P15, and P16). This was performed before the development of malignancy or any clinical manifestations (25–27).

Preemptive HSCT shows promise in terms of immune reconstitution. However, patients with ataxia telangiectasia (A-T) often receive a diagnosis at a rather late due to the manifestation of telangiectasia after the age of three. There is a significantly increased likelihood of presenting with malignancy in the long term, with a risk being approximately 30 to 70 times higher than that of the general population (29, 30). In this context, current treatment strategies and ongoing discussions are particularly important for A-T patients who present with malignancies. Malignancy continues to be the primary cause of long-term mortality among A-T patients. However, due to the high toxicity associated with conditioning regimens, routine use of HSCT is not currently recommended (1, 8). With the exception of our patient, five A-T patients with malignancy have been treated with a RIC regimen. Unfortunately, only two of these patients survived. Achieving the optimal balance to effectively treat malignant cells in A-T patients without excessive adverse effects and toxicity constitutes a significant therapeutic challenge. One such patient is a 13-year-old A-T patient with EBV-related non-Hodgkin lymphoma (P5) (21). While Beier et al. reported that the patient survived disease-free, there were life-threatening complications, including hepatic veno-occlusive disease, gastrointestinal bleeding, recurrent sepsis, and the need for ventilatory support with the potential for long-term sequelae. Another patient (P2), reported as alive by Ussowicz et al., experienced drug-related toxicities including grade 4 chronic leucopenia (0.25 K/mL) with agranulocytosis (0.13 K/mL) and mucositis after transplantation. This patient required repeated donor lymphocyte infusions and additional treatments (25). However, the other patients (P12, P13, P14) succumbed to graft-versus-host disease (GvHD), drug-related toxicities, and multiorgan failure (23). Therefore, it is essential to prioritize the prevention of GVHD without compromising the graft-versus-tumor/leukemia effect of T- and NK-lymphocytes within the graft. Conventional treatment protocols for malignant diseases involving radiotherapy and/or chemotherapy carry a high risk of therapy-related morbidity and mortality in these patients. In contrast to other cases, our patient was treated with a conditioning protocol that included TDM of the main alkylating agent to optimize myeloablation and the administration of rabbit anti-thymocyte globulin to achieve the aforementioned goals. After thorough observation, we can confirm that there was no evidence of toxicity or GvHD. The administration of only a reduced dose of treosulfan (1x12 gr/m2) has been shown to achieve the target cumulative AUC. This approach has resulted in a tailored, well-controlled myeloablation without significant toxicity and has led to a leukemia-free survival after 3 years post-HSCT. Although TDM for treosulfan is not yet a standard practice or formally endorsed, our reports underscore its relevance and practicality when treating malignant diseases associated with DNA repair and chromosomal instability syndromes.

Given the poor overall survival rate of transplanted A-T patients in the past, a new discussion is mandatory regarding the potential of HSCT in patients with A-T. This discussion should include contemporary approaches, such as TDM for treosulfan, *in-vivo* T-cell depletion and/or drug profiling tests. These tests can help identify effective anti-leukemic drugs with lower toxicity, such as ponatinib and venetoclax. These approaches have the potential to achieve long-term remissions and relapse-free survival after leukemias and lymphomas. Our case also demonstrates that long-term immune-reconstitution of impaired B- and T-cells, including *de-novo* production of recent thymic emigrants after thymic reactivation, can be achieved in A-T patients. This suggests the potential for further refinement of novel options and modifications of HSCT for the treatment of both malignancies and of combined immunodeficiency, thereby enriching the repertoire of treatment regimens for A-T patients.

## Data Availability

The original contributions presented in the study are included in the article. Further inquiries can be directed to the corresponding author.
